# Isolated Goutallier Grade 4 supraspinatus atrophy: a rare case without rotator cuff tear or nerve compression

**DOI:** 10.1007/s00256-026-05280-6

**Published:** 2026-06-12

**Authors:** Trisha Mukherjee, Arash D. Tehranzadeh, John M. Itamura, Brian K. Lee

**Affiliations:** 1 Creighton University School of Medicine, Phoenix, AZ USA; 2 Department of Radiology, Cedars-Sinai Orthopaedics, Los Angeles, CA USA; 3 Department of Orthopaedics, Cedars-Sinai Orthopaedics, Los Angeles, CA USA

**Keywords:** Rotator cuff, Shoulder, Magnetic resonance imaging, Supraspinatus muscle atrophy, Suprascapular nerve

## Abstract

The supraspinatus muscle is susceptible to atrophy, with fatty infiltration most commonly attributed to chronic full-thickness tendon tears. Chronic denervation due to suprascapular nerve entrapment may also be responsible for supraspinatus atrophy. However, such cases typically show concurrent supraspinatus and infraspinatus involvement due to the muscles’ shared innervation by the suprascapular nerve. We report a rare case of isolated supraspinatus atrophy in a 66-year-old male with intact rotator cuff tendons, preserved infraspinatus morphology, and no evidence of tendon tear or nerve compression lesion. The patient presented with gradually worsening anterior shoulder pain that began following a workout injury, without accompanying weakness or neurological symptoms. Magnetic resonance imaging revealed Goutallier Grade 4 fatty atrophy of the supraspinatus muscle and advanced acromioclavicular joint arthritis. Given the absence of typical structural etiologies, a chronic subclinical denervation pattern was considered. Two plausible mechanisms were explored: (1) selective injury of an early-branching motor division of the suprascapular nerve supplying the supraspinatus, and (2) an atypical manifestation of Parsonage-Turner syndrome, both of which can lead to isolated supraspinatus denervation without overt clinical deficits. To the best of our knowledge, this represents only the second documented case of isolated supraspinatus atrophy without rotator cuff disruption, nerve compression, or infraspinatus involvement. The case reinforces the diagnostic value of magnetic resonance imaging and highlights the importance of considering neural etiologies in cases where imaging findings deviate from classic rotator cuff pathology.

## Introduction

The supraspinatus muscle is a key component of the rotator cuff, contributing to shoulder abduction and dynamic glenohumeral stability, and is particularly prone to atrophy [1, 2]. The Goutallier classification system is used in clinical practice to quantify fatty infiltration, with Grade 4 indicating near-complete fatty replacement [3]. In the supraspinatus, severe atrophy is most commonly associated with chronic full-thickness tendon tears, often accompanied by tendon retraction and irreversible muscle volume loss [4]. However, atrophy may also result from chronic denervation, frequently due to entrapment of the suprascapular nerve at the suprascapular or spinoglenoid notch [5–7]. When the suprascapular nerve is compressed at the suprascapular notch, both the supraspinatus and infraspinatus muscles typically demonstrate atrophy due to their shared innervation [6–9]. Conversely, nerve compression at the spinoglenoid notch typically results in isolated infraspinatus atrophy with preservation of the supraspinatus muscle due to proximal innervation of the supraspinatus relative to the infraspinatus [6–9].

The presence of isolated Grade 4 supraspinatus atrophy, in the absence of tendon disruption, retraction, infraspinatus involvement, or radiographic signs of nerve entrapment deviates from typical imaging associations [4, 8, 10]. Leclère et al. was the first to report such a case, describing a patient with Goutallier Grade 4 supraspinatus atrophy despite having intact rotator cuff tendons and no identifiable nerve compression or traction-based etiology [10]. Their findings suggested a neurogenic mechanism as the most plausible explanation, emphasizing that profound muscle atrophy and volume loss can occur even in the absence of overt structural pathology or visible nerve compression [10].

We present, to the best of our knowledge, the second documented case of isolated Goutallier Grade 4 supraspinatus atrophy, with an otherwise intact rotator cuff. The patient demonstrated no evidence of tendon retraction, full-thickness tear, or nerve compression lesion, and the infraspinatus exhibited preserved morphology.

Building upon the observations of Leclère et al., our case explores two plausible neurogenic mechanisms that may explain this uncommon presentation: (1) a selective injury of an early-branching motor division of the suprascapular nerve supplying the supraspinatus; and (2) an atypical presentation of Parsonage-Turner syndrome (PTS) [10]. Both mechanisms reflect subtle forms of denervation that may not manifest on clinical examination, particularly in the absence of pain, sensory changes, or overt muscle imbalance, but can result in profound muscle atrophy detectable on magnetic resonance imaging (MRI) [11–13].

This case underscores the importance of recognizing atypical neurogenic patterns of rotator cuff atrophy that may mimic chronic tendon pathology on imaging and highlights how MRI can aid in identifying these patterns and guide more accurate diagnosis and treatment planning.

### Case report

A 66-year-old male with no significant past medical or surgical history presented with anterior left shoulder pain of approximately six weeks’ duration. The pain began insidiously during a workout and progressively interfered with his ability to continue exercising. The patient reported no constitutional or neurological symptoms and had not yet initiated physical therapy at the time of presentation. His medical history was notable only for cervical arthritis.

On initial physical examination, the patient was well developed and in no acute distress. There were no skin abnormalities or visible atrophy of the upper extremities. Range of motion of the left shoulder was mildly reduced, with forward elevation to 150°, external rotation at 90° abduction to 80°, and internal rotation to T12, while passive motion was preserved. Rotator cuff tests for supraspinatus, infraspinatus, and subscapularis were negative for both pain and weakness. Speed’s, Yergason’s, and cross-body adduction tests were positive. Sensation and neurovascular status of the upper extremities were intact. Radiographs of the left shoulder showed no fractures or subluxations, and the glenohumeral and acromioclavicular joint demonstrated no signs of instability. The patient was referred to physical therapy and was provided a referral for a left shoulder MRI without contrast.

**Fig. 1:**
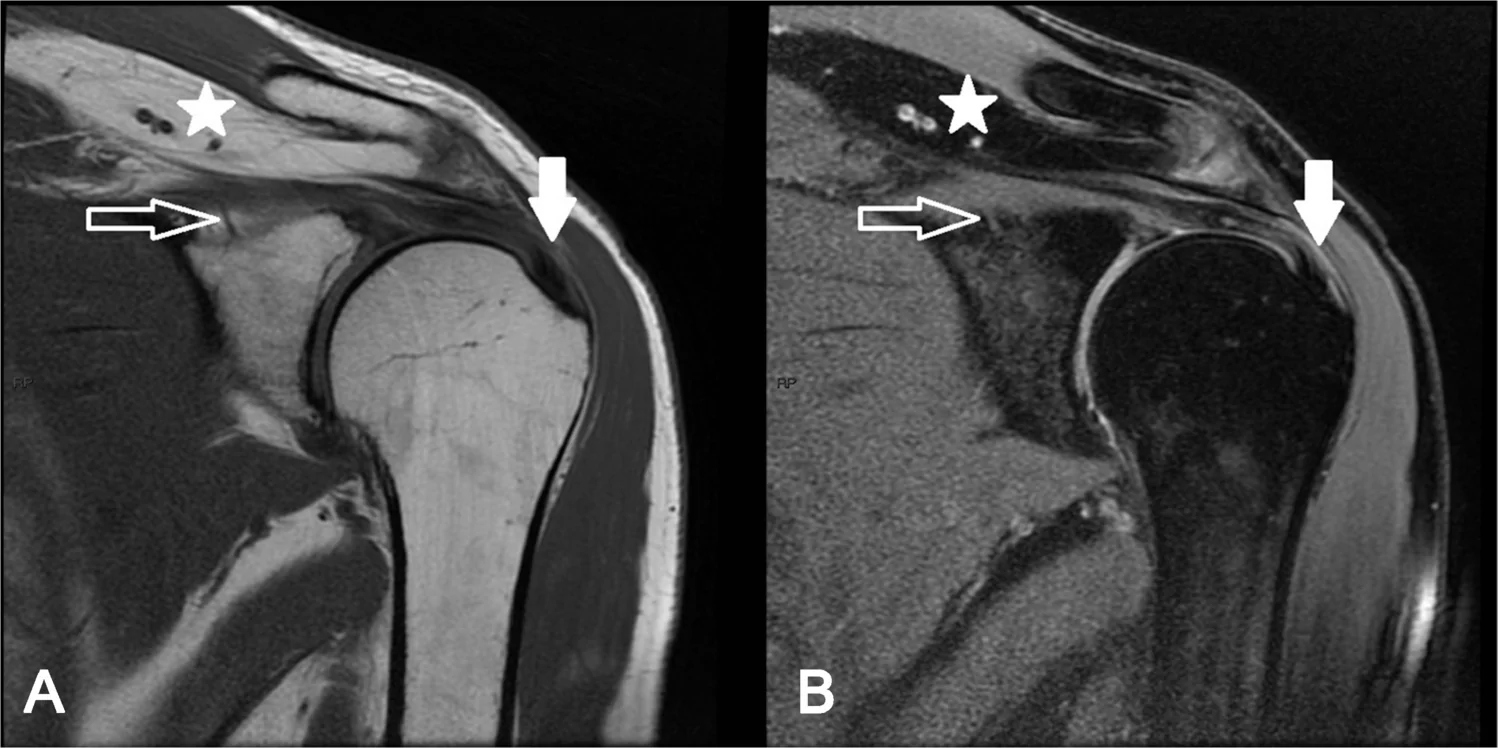
MR of the shoulder at the Suprascapular notch. Coronal oblique T1-weighted image (**A**) and corresponding Proton Density with Fat Saturation (**B**) demonstrates normal morphology and signal of the suprascapular nerve at the suprascapular notch (white open arrow). Adjacent severe fatty atrophy of the supraspinatus muscle (white solid star) is present and the intact distal supraspinatus tendon insertion shows mild tendinosis (white solid arrow)

**Fig. 2:**
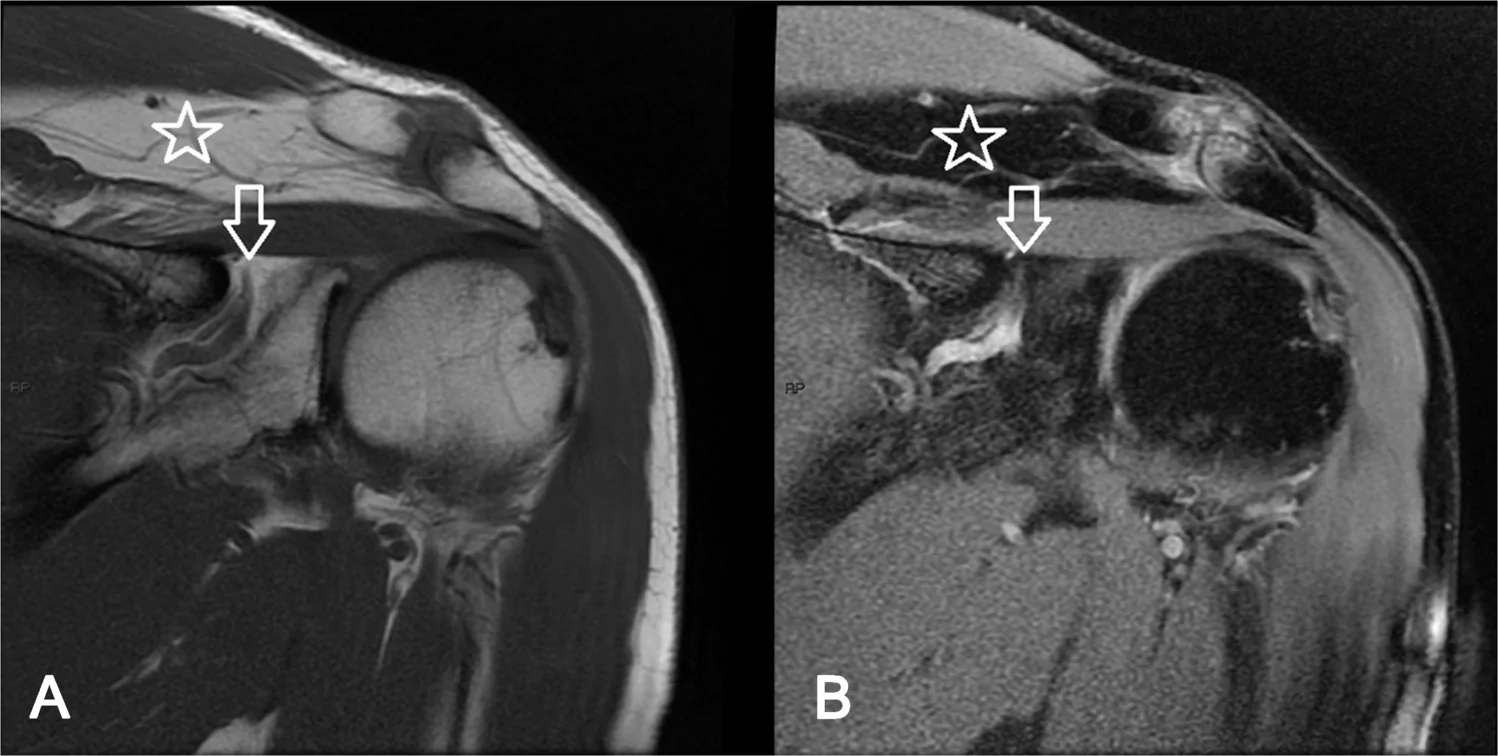
MR of the shoulder at the Spinoglenoid notch. Coronal oblique T1-weighted image (**A**) and corresponding Proton Density with Fat Saturation (**B**) demonstrates normal morphology and signal of the neurovascular bundle as it descends into the spinoglenoid notch (white open arrow). Severe fatty atrophy of the supraspinatus muscle is illustrated (white open star)

**Fig. 3:**
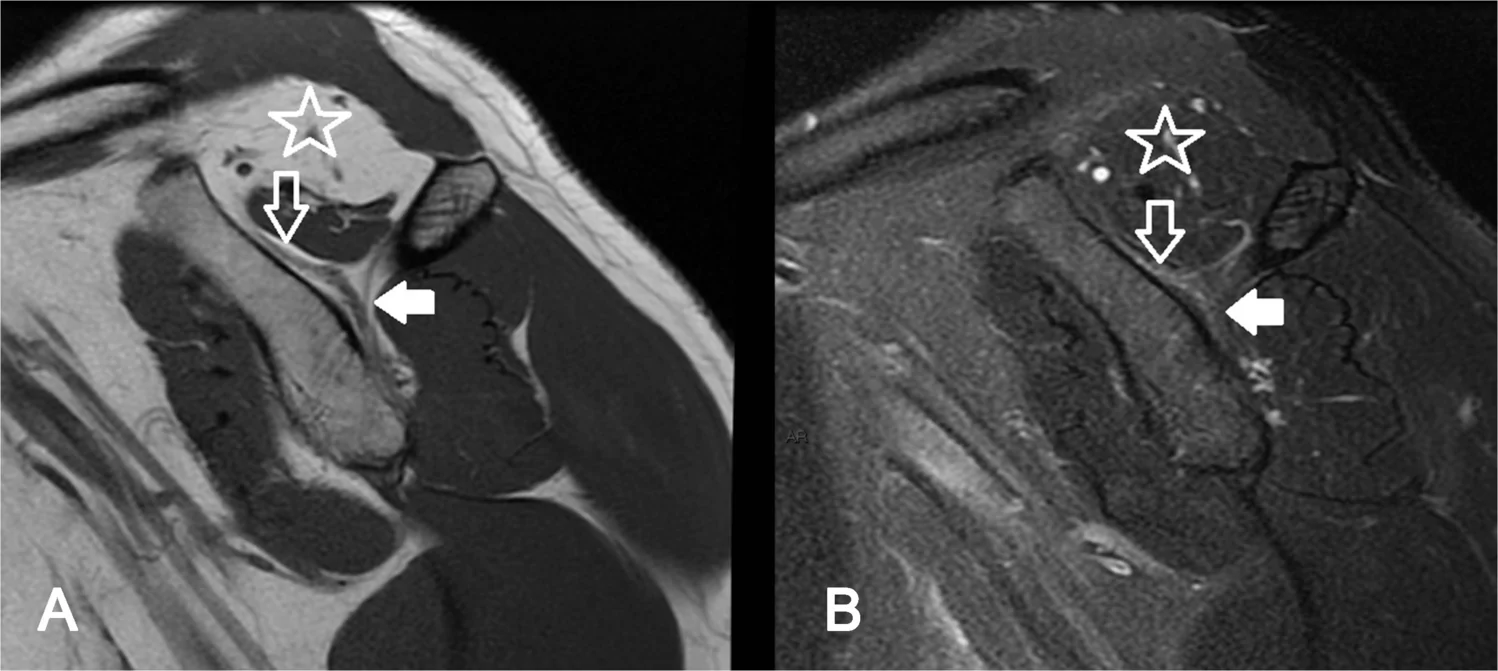
MR of the Shoulder at the Suprascapular and Spinoglenoid notch. Sagittal oblique T1-weighted image (**A**) and corresponding T2 with Fat Saturation (**B**) displays the nerve with normal morphology and signal, without intrinsic or extrinsic mass, as it courses through the level of the suprascapular notch (white open arrow) and its distal passage and descent through the level of the spinoglenoid notch (white solid arrow). Severe atrophy and decreased muscle volume of the supraspinatus muscle are documented (white open star)

MRI performed three months after symptom onset demonstrated severe acromioclavicular joint arthritis with subchondral cyst formation and joint inflammation. There was Goutallier Grade 4 fatty atrophy and decreased volume of the supraspinatus muscle with an intact distal tendon insertion demonstrating mild tendinosis (Figs. 1 and 2). The infraspinatus muscle appeared preserved without evidence of fatty infiltration (Fig. 3). Evaluation of the suprascapular and spinoglenoid notches demonstrated normal morphology and signal of the suprascapular nerve without evidence of focal nerve compression, ganglion, or mass lesion (Figs. 1–3). No advanced glenohumeral joint osteoarthritis was observed.

At this follow-up visit, the patient reported residual anterior shoulder discomfort despite adherence to physical therapy, although his symptoms had improved overall. Examination revealed reduced left shoulder forward elevation to 140° and internal rotation to T6, with other ranges of motion preserved. Rotator cuff testing remained negative for weakness or pain. Cross-body adduction reproduced his anterior shoulder pain. Neurologic and motor function, including deltoid activation and hand dexterity, were intact.

Treatment options were discussed, including continued physical therapy, diagnostic lidocaine injection, corticosteroid injection, platelet-rich plasma, and potential surgical intervention. Additional diagnostic testing, including electromyography, was also discussed; however, given overall improvement in symptoms, the patient elected to continue conservative management and defer further diagnostic evaluation at that time.

## Discussion

This case presents an uncommon imaging pattern: Goutallier Grade 4 supraspinatus atrophy, occurring without a full-thickness tendon tear, tendon retraction, nerve compression lesion, or infraspinatus involvement. Notably, severe acromioclavicular joint arthritis was also present, with subchondral cyst formation and joint inflammation. Given the chronic appearance of the supraspinatus fatty atrophy and the subacute onset of symptoms, tenderness to palpation at the acromioclavicular joint, and pain with cross body test, the acromioclavicular joint pathology was considered a more plausible primary pain generator, with the supraspinatus finding serving as an incidental yet diagnostically important imaging observation.

This pattern deviates from the typical features of chronic rotator cuff pathology resulting from tendon tearing and retraction [3, 4, 14]. It also differs from the characteristic presentation of suprascapular nerve compression, which more often results in combined supraspinatus and infraspinatus atrophy, due to shared innervation from the suprascapular nerve [1, 2, 5, 6, 8]. In this case, MRI demonstrated a normal U-shaped suprascapular notch rather than a V- or W-shaped variant, and no thickening or calcification of the superior transverse scapular ligament, which are morphologies known to increase the risk of nerve entrapment [7, 15]. The absence of both a rotator cuff tear and imaging features suggestive of nerve entrapment supports a chronic subclinical neurogenic process, likely reflecting selective denervation of motor branches to the supraspinatus that progressed over time [2, 12].

One plausible explanation for these findings is anatomical variation in the branching pattern of the suprascapular nerve [6, 7]. Cadaveric studies have demonstrated that in some individuals, the motor branches to the supraspinatus may originate proximal to the suprascapular notch, rather than within the supraspinous fossa [16]. Irrespective of their origin, these branches are generally fewer in number and thinner in caliber, characteristics that make them more susceptible to traction or stretch-related injury [16]. This vulnerability is further amplified when the branches arise proximally, as their longer and less protected course increases exposure to mechanical strain during shoulder motion [16, 17].

Importantly, even when originating proximally, these motor fibers must still traverse or closely pass by the suprascapular notch to reach the supraspinatus [16]. This mechanically vulnerable region is subject to repetitive strain, which may induce isolated denervation in anatomically predisposed individuals [16–18]. This phenomenon, termed *microdynamic entrapment*, refers to a non-compressive form of neural irritation driven by chronic mechanical stress and has been proposed as a mechanism of suprascapular neuropathy among individuals with structural or positional vulnerabilities [17, 18]. In this patient, known acromioclavicular joint arthritis may have altered scapulothoracic mechanics, thereby increasing local strain at the suprascapular notch [1, 7]. Combined with a long-standing history of exercise-related shoulder loading, these factors may have imposed chronic tension on anatomically vulnerable motor branches, ultimately resulting in the isolated supraspinatus atrophy observed on imaging.

An alternative diagnostic consideration is PTS, an idiopathic brachial plexopathy typically characterized by acute onset shoulder pain followed by progressive muscle weakness and atrophy [11, 19, 20]. Although the classic presentation involves abrupt, severe pain, atypical cases with minimal or absent pain are well-documented [20, 21]. Additionally, the suprascapular nerve is among the most commonly affected, involved in over 70% of reported cases [21, 22]. While PTS often follows a viral illness or recent vaccination, post-traumatic and exertion-related triggers have also been described [19, 21]. In this patient, the absence of a structural lesion, the chronic denervation changes isolated to the supraspinatus, and the long-standing history of exercise-related shoulder loading support the consideration of an atypical variant of PTS [21, 22]. Large cohort studies have shown that PTS can affect nerves in a patchy or isolated manner, making it a plausible explanation for selective involvement of the motor branches to the supraspinatus, particularly given their previously discussed anatomic vulnerability [11, 16, 21, 22].

Without electrodiagnostic testing, the presence and extent of supraspinatus denervation could not be confirmed in this patient. Electromyography may have helped identify a neural etiology and assess for subclinical involvement of additional muscles or nerves, which could have supported a multifocal process such as PTS [11, 13, 20]. In the absence of such findings, differentiation between an atypical presentation of PTS and a selective suprascapular nerve branch injury related to anatomical variation remains challenging [11, 12, 19]. Additional imaging techniques, including cervical spine MRI and magnetic resonance neurography of the brachial plexus, may further aid in distinguishing between these potential etiologies through more detailed assessment of the suprascapular nerve throughout its course [23].

This case highlights a rare presentation of Goutallier Grade 4 supraspinatus atrophy occurring without rotator cuff tear, tendon retraction, nerve compression lesion, or infraspinatus involvement. Recognition of this imaging pattern may prompt consideration of subtle neurogenic etiologies, including selective suprascapular nerve branch injury or atypical PTS.

## Data Availability

Not applicable.
